# Replication of Porcine Astrovirus Type 1-Infected PK-15 Cells *In Vitro* Affected by RIG-I and MDA5 Signaling Pathways

**DOI:** 10.1128/spectrum.00701-23

**Published:** 2023-05-04

**Authors:** Qinting Dong, Xinyue Zhu, Leping Wang, Wenchao Zhang, Lifei Lu, Jun Li, Shuhong Zhong, Chunxia Ma, Kang Ouyang, Ying Chen, Zuzhang Wei, Yifeng Qin, Hao Peng, Weijian Huang

**Affiliations:** a Laboratory of Animal Infectious Diseases and Molecular Immunology, College of Animal Science and Technology, Guangxi University, Nanning, China; b Guangxi Key Laboratory of Veterinary Biotechnology, Guangxi Veterinary Research Institute, Nanning, Guangxi, China; c Guangxi Zhuang Autonomous Region Engineering Research Center of Veterinary Biologics, Nanning, China; d Guangxi Key Laboratory of Animal Reproduction, Breeding and Disease Control, Nanning, China; e Key Laboratory of China (Guangxi)-ASEAN Cross-Border Animal Disease Prevention and Control, Ministry of Agriculture and Rural Affairs of China, Nanning, China; f Guangxi Colleges and Universities Key Laboratory of Prevention and Control for Animal Disease, Nanning, China; Geisel School of Medicine at Dartmouth

**Keywords:** PAstV, interferon, viral replication, RIG-I, MDA5

## Abstract

The interferon (IFN) system is an extremely powerful antiviral response in animal cells. The subsequent effects caused by porcine astrovirus type 1 (PAstV1) IFN activation are important for the host’s response to viral infections. Here, we show that this virus, which causes mild diarrhea, growth retardation, and damage of the villi of the small intestinal mucosa in piglets, induces an IFN response upon infection of PK-15 cells. Although IFN-β mRNA was detected within infected cells, this response usually occurs during the middle stages of infection, after genome replication has taken place. Treatment of PAstV1-infected cells with the interferon regulatory factor 3 (IRF3) inhibitor BX795 decreased IFN-β expression, whereas the nuclear factor kappa light chain enhancer of activated B cells (NF-κB) inhibitor BAY11-7082 did not. These findings indicate that PAstV induced the production of IFN-β via IRF3-mediated rather than NF-κB-mediated signaling pathways in PK-15 cells. Moreover, PAstV1 increased the protein expression levels of retinoic acid-inducible gene I (RIG-I) and melanoma differentiation-associated protein 5 (MDA5) in PK-15 cells. The knockdown of RIG-I and MDA5 decreased the expression levels of IFN-β and the viral loads and increased the infectivity of PAstV1. In conclusion, PAstV1 induced the production of IFN-β via the RIG-I and MDA5 signaling pathways, and the IFN-β produced during PAstV1 infection inhibited viral replication. These results will help provide new evidence that PAstV1-induced IFNs may protect against PAstV replication and pathogenesis.

**IMPORTANCE** Astroviruses (AstVs) are widespread and can infect multiple species. Porcine astroviruses produce mainly gastroenteritis and neurological diseases in pigs. However, astrovirus-host interactions are less well studied, particularly with respect to their antagonism of IFN. Here, we report that PAstV1 acts via IRF3 transcription pathway activation of IFN-β. In addition, the knockdown of RIG-I and MDA5 attenuated the production of IFN-β induced by PAstV1 in PK-15 cells and increased efficient viral replication *in vitro*. We believe that these findings will help us to better understand the mechanism of how AstVs affect the host IFN response.

## INTRODUCTION

Astroviruses (AstVs) belong to the family *Astroviridae* and infect over 80 avian (*Avastrovirus*) and mammalian (*Mamastrovirus*) host species (1). Human AstVs (HAstVs) cause viral gastroenteritis worldwide, and they are the third most common cause of infection in the pediatric population after rotaviruses and noroviruses (2). In addition to children, HAstV gastroenteritis also commonly occurs in the elderly (3) and in immunocompromised individuals (4, 5). Additionally, in recent years, more serious diseases such as enteritis and those with neurological symptoms have been found in humans and other mammalian species (6–10). So far, recombination events involving human strains have been suggested to have occurred between classical HAstV and a California sea lion AstV (11) as well as between classical HAstV and porcine astrovirus (PAstV) (12). However, little is known regarding their impact on the molecular mechanisms of the interferon (IFN) response to AstV replication.

The AstV genome is a small, single-stranded, nonenveloped, positive-sense RNA comprising approximately 6.2 to 7.9 kb. The genomic RNA (gRNA) consists of a 5′ untranslated region (UTR), three overlapping open reading frames (ORFs) (ORF1a, ORF1b, and ORF2), a 3′ UTR, and a poly(A) tail (13). ORF1a and ORF1b encode nonstructural proteins (NSPs) involved in RNA transcription and replication, and ORF2 encodes structural proteins that play essential roles in virus entry (14). More recently, ORFX of different lengths was detected in nearly all astrovirus sequences from humans and other mammalian species (15, 16). In 1980, PAstVs were first reported in pigs showing diarrheal symptoms (17). PAstVs have been reported globally, and they were considered to be associated with diarrhea and neurological diseases (9, 18, 19). They are divided into at least five distinct lineages, PAstV1 to PAstV5, based on their ORF2 sequences (20–22), and all of these genotypes were found to be prevalent in China (23). PAstV1 to PAstV5 are widespread in the pig population, and in recent years, incidences of coinfections with both dual and triple genotypes have also been shown to occur (22, 24). The results of genetic evolutionary analyses suggest that PAstV may have crossed the species barrier between humans and other animals. Several lines of evidence suggest that the interspecies barrier for PAstV may not be strict (12, 25, 26).

The innate immune system forms the first line of defense against invading viruses, limiting their initial replication and ensuring the survival of the host until a complete and specific response is developed. The IFN system is an extremely powerful antiviral response that is capable of dealing with most viral infections in the absence of adaptive immunity (27). The retinoic acid-inducible gene I (RIG-I)-like receptors (RLRs) in host cells are able to sense the viral component, and they play an essential role in the production of type I IFN as well as proinflammatory cytokines (27). In recent studies, it was shown that HAstVs induced a mild and delayed interferon response upon infection of CaCo-2 cells, and viral replication could be partially reduced by the addition of exogenous IFN (28). Additionally, innate immune responses were shown to contribute to the control of viral replication in infected turkeys and mice (29, 30). Previous studies have also shown that for numerous RNA viruses, RIG-I is responsible for detecting negative-strand RNA viruses such as paramyxovirus, influenza virus, and vesicular stomatitis virus (VSV) as well as two related positive-strand viruses, Japanese encephalitis virus and hepatitis C virus (HCV) (31–33). Melanoma differentiation-associated protein 5 (MDA5) has also been shown to mediate the recognition of another positive-strand RNA virus, encephalomyocarditis virus, which is a prototypic member of the picornavirus family (32, 34). Dengue virus (DENV) can be recognized by the RIG-I/MDA5 system, and this induces the production of IFNs (35). In addition, both RIG-I and MDA5 function in a cooperative manner to establish an antiviral state in response to West Nile Virus (WNV) infection (36). Therefore, in this study, PAstVs were shown to stimulate IFN production in host cells, and the role of RIG-I and MDA5 in PAstV-induced IFN production was investigated. To further understand the relationship between PAstV replication and IFN production during infection, viral replication was studied after the knockdown of RIG-I and MDA5.

## RESULTS

### PAstV1 induced the production of IFN-β in PK-15 cells.

**FIG 1:**
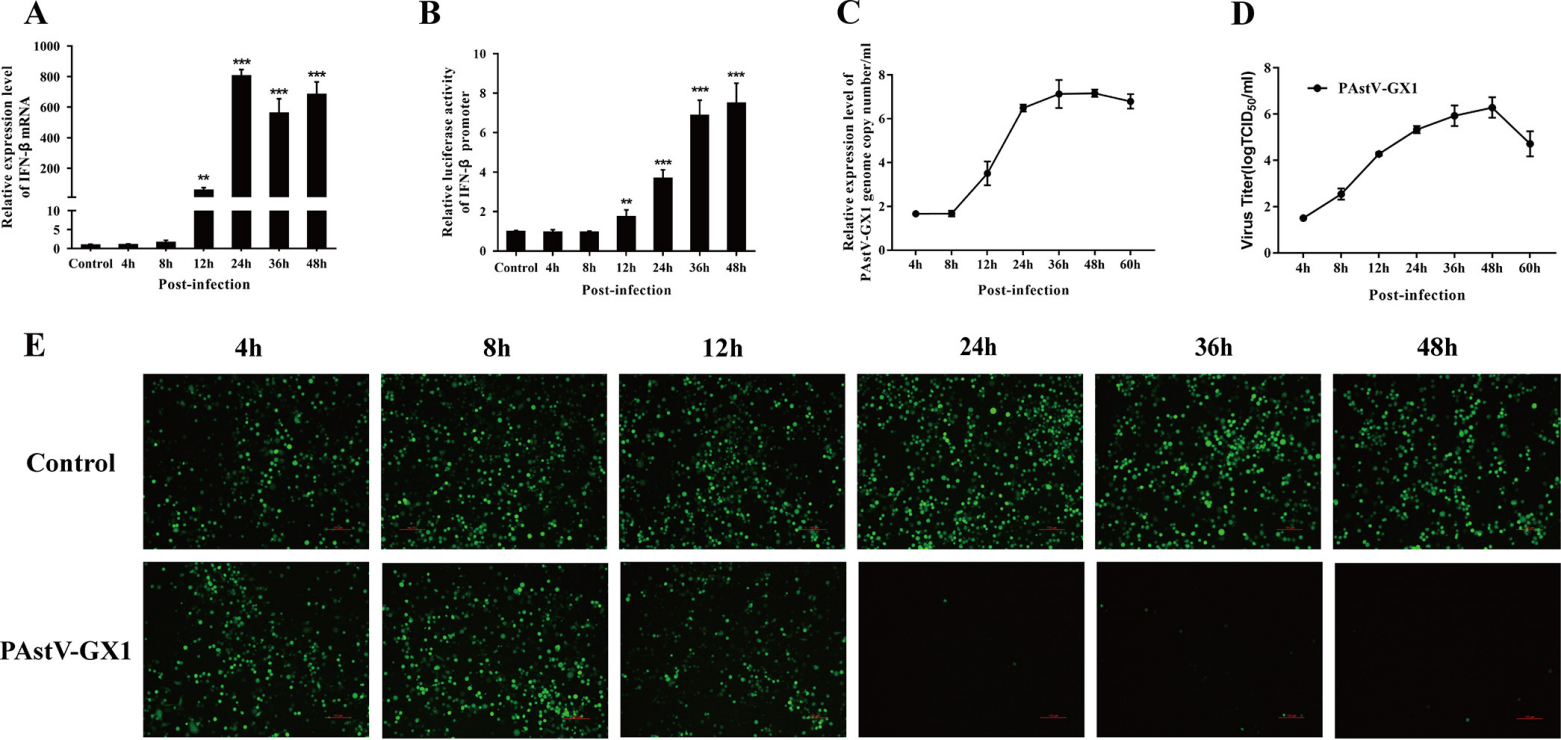
Similar trends in PAstV-GX1 replication and IFN-β expression induced in PK-15 cells. (A and B) Quantitative real-time PCR (A) and luciferase analysis (B) were used to measure the mRNA expression levels of IFN-β and the activation levels of the IFN-β promoter in both control cells and PK-15 cells infected with PAstV-GX1 at an MOI of 0.01. (C) The cell culture supernatants were harvested after infection at the indicated times and assayed for the production of infectious virus by a TCID_50_ assay on PK-15 cells. The TCID_50_ per milliliter were calculated using the Reed-Muench formula. Each data point represents the average titer derived from three independent TCID_50_ assays. (D) The total RNA was isolated after PAstV1 infection at the indicated times. The PAstV-GX1 genome copy number growth curve was determined by RT-PCR. (E) PK-15 cells infected with PAstV-GX1 at an MOI of 0.01. After infection, the cell supernatants were harvested. Following UV irradiation, the harvested cell supernatants were overlaid onto fresh PK-15 cells in 24-well plates. Twenty-four hours after treatment, the cells were infected with VSV-GFP, and 24 h after infection, virus replication was determined by fluorescence microscopy. The results shown are representative of data from three independent experiments. **, *P* < 0.01; ***, *P* < 0.001.

In order to investigate whether PAstV1 induces the production of IFN-β, quantitative real-time PCR (qPCR) and an IFN-β promoter luciferase reporter system were used to measure the mRNA levels and promoter activities of IFN-β in PK-15 cells after infection with PAstV-GX1 at a multiplicity of infection (MOI) of 0.01 for 4, 8, 12, 24, 36, and 48 h. The results showed that the mRNA expression levels of IFN-β in the PAstV1-infected group at 12 h were significantly higher than those in the control group (*P* < 0.01) (Fig. 1A). Similar results were also observed for the promoter activity levels determined by using the IFN-β promoter luciferase reporter system. The IFN-β promoter activities in the PAstV1-infected group at 12 h were also markedly increased compared with those in the control group at multiple infection cycles (*P* < 0.05) (Fig. 1B). To further confirm the results from these qPCR and IFN-β promoter luciferase reporter assays, we performed IFN bioassays by using an IFN-sensitive vesicular stomatitis virus expressing green fluorescent protein (VSV-GFP). The level of VSV-GFP replication is inversely linked to the levels of IFN-α/β secreted from infected PK-15 cells. As shown in Fig. 1E, the cellular supernatants from PAstV-1-infected cells at 24 h significantly inhibited the replication of VSV-GFP in PK-15 cells compared with the supernatants from control cells. In addition, in the multiple-infection-cycle model (MOI = 0.01), the virus gRNA was increased at 12 h and accumulated at 36 to 48 h postinfection (hpi) (Fig. 1D). However, the virus titer reached 2 × 10^6^ 50% tissue culture infective doses (TCID_50_)/mL at 24 h, peaked at 48 h, and then decreased gradually (Fig. 1C). These results demonstrated that PAstV1 induced IFN-β production in PK-15 cells and that rapid viral replication occurred before IFN-β expression.

### PAstV1 induced the production of IFN-β via IRF3 rather than NF-κB in PK-15 cells.

**FIG 2:**
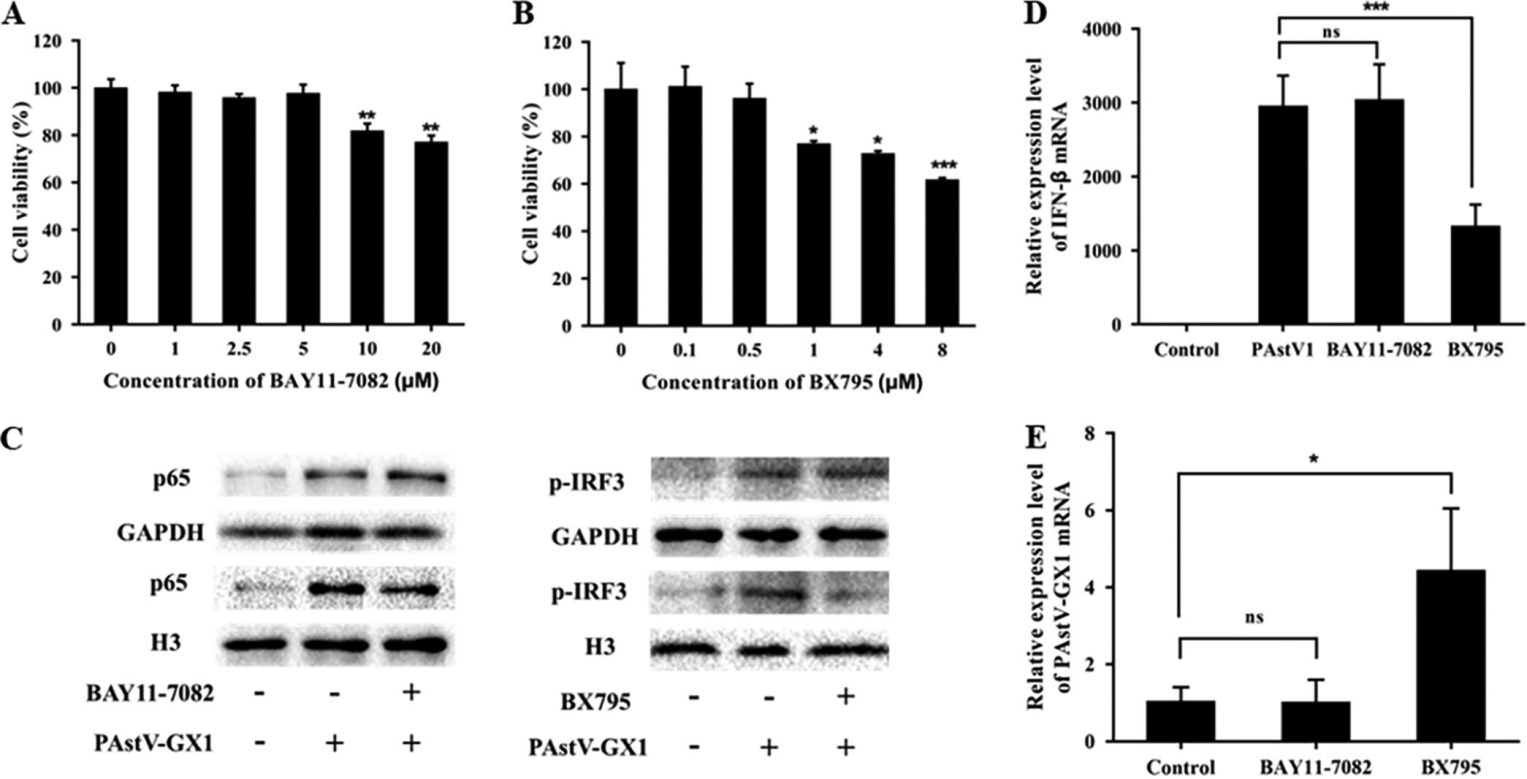
Effects of BAY11-7082 and BX795 on PK-15 cell viability and mRNA expression levels of IFN-β in PAstV-infected PK-15 cells. (A and B) PK-15 cells were treated with different concentrations of BAY11-7082 (A) and BX795 (B) for 48 h, and an MTT assay was used to assess cell viability. (C) PK-15 cells were treated with either 5 μM BAY11-7082 or 0.5 μM BX795 and infected with PAstV-GX1 at an MOI of 0.01 for 24 h. Western blotting was performed to measure the protein levels of p65 and p-IRF3 in the nucleus and cytoplasm, respectively. (D and E) The mRNA levels of IFN-β (D) and PAstV-GX1 (E) were measured by quantitative real-time PCR. The results shown are representative of data from three independent experiments. *, *P* < 0.05; ***, *P* < 0.001; ns, not significant.

The activation of the transcription factors NF-κB and interferon regulatory factor 3 (IRF3) plays an important role in the production of IFN-β. To determine which transcription factor was associated with IFN-β production induced by PAstV1 infection in PK-15 cells, the inhibitors BAY11-7082 and BX795 were used. BAY11-7082 inhibits NF-κB expression by blocking IκBα phosphorylation, while BX795 inhibits IRF3 activation by blocking the catalytic activities of Tank-binding kinase 1 (TBK1) and IκB kinase ε (IKKε) (37). MTT [3-(4,5-dimethyl-2-thiazolyl)-2,5-diphenyl-2H-tetrazolium bromide] analysis showed that there was no associated toxicity in PK-15 cells at 5 μM and 0.5 μM BAY11-7082 and BX795 at 72 h, respectively (Fig. 2A and B). Based on these results, PK-15 cells were incubated with either 5 μM BAY11-7082 or 0.5 μM BX795 and then infected with PAstV-GX1 at an MOI of 0.01 for 24 h. The p65 and phosphorylated IRF3 (p-IRF3) protein levels were then determined by Western blotting. The nuclear protein levels of p65 in the BAY11-7082-treated group and the cytosolic levels of p-IRF3 in the BX795-treated group were significantly reduced compared to those in the PAstV1-infected group, indicating that the inhibitors blocked the activation of NF-κB and IRF3, respectively (Fig. 2C). qPCR was used to determine the expression levels of IFN-β and PAstV-GX1 mRNAs. The results showed that the expression of IFN-β mRNA was significantly reduced in the BX795-treated group and that the expression of PAstV-GX1 mRNA was significantly induced in the BX795-treated group compared to the control group (Fig. 2D and E). Furthermore, there was no significant difference in the expression levels of IFN-β and PAstV-GX1 mRNAs between the BAY11-7082 treatment group and the control group (Fig. 2D and E). These results suggested that PAstV1 induced IFN-β production via the IRF3- rather than the NF-κB-mediated signaling pathway.

### PAstV1 induced the mRNA expression of ISG15, ISG56, RIG-I, and MDA5 in PK-15 cells.

**FIG 3:**
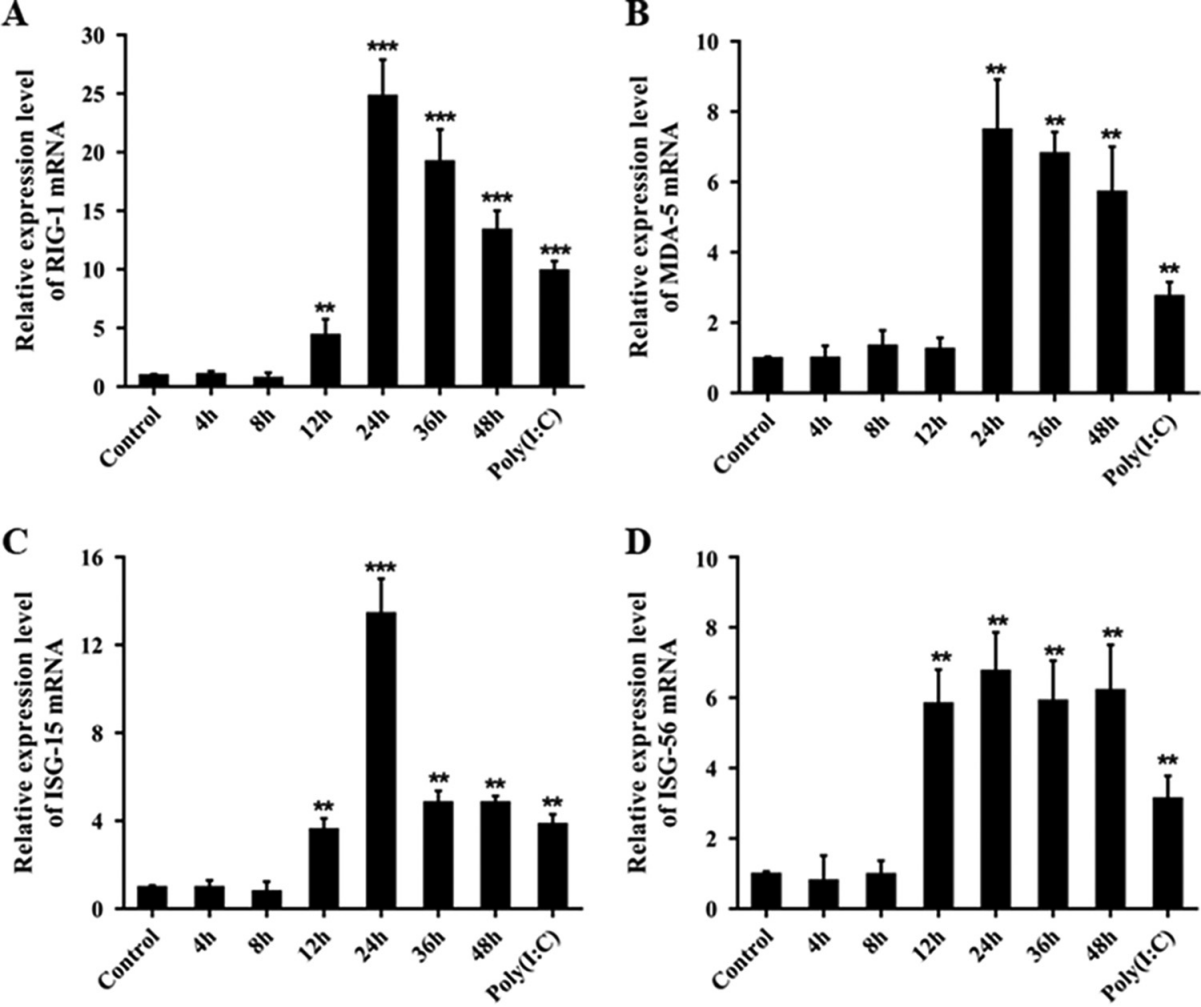
PAstV infection induced by ISG expression. PK-15 cells were seeded in 12-well plates and then infected with PAstV1 at an MOI of 0.01, and cells treated with 1 μg/mL poly(I·C) were used as a positive control. Total RNA was isolated at the indicated times after PAstV1 infection. RT-PCR was employed to measure the relative RNA expression levels. Fold changes in the expression of RIG-I (A), MDA5 (B), ISG15 (C), and ISG56 (D) are shown. The data are the means and standard deviations from three independent experiments. *, *P* < 0.05; **, *P* < 0.01.

Due to the similar trends in the rapid replication of viral gRNA and IFN-β, it appeared that IFN-β failed to inhibit PAstV infection. To determine whether IFN-β exerted antiviral activity during PAstV1 infection, we examined the expression levels of some IFN-stimulated genes (ISGs). The mRNAs of RIG-I (Fig. 3A), MDA5 (Fig. 3B), ISG15 (Fig. 3C), and ISG56 (Fig. 3D) were all upregulated following high-level expression of IFN-β, and the levels were higher than those in the poly(I·C)-treated group. This suggested that the type I IFN signaling pathway was not blocked by PAstV1 infection. Interestingly, the mRNA levels of RIG-I, ISG15, and ISG56 were transiently upregulated 12 h after PAstV1 infection, while those of MDA5 were significantly elevated at 24 h. These results suggested that PAstV1 infection of PK-15 cells at 12 h induced the innate antiviral immune response and that the activation of the IFN response required an abundance of viral gRNA.

### PAstV1 induced the activation of RIG-I and MDA5 in PK-15 cells.

**FIG 4:**
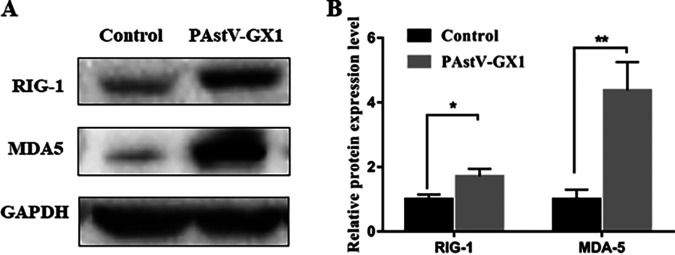
Changes in the protein expression levels of RIG-I and MDA5 in PAstV-infected PK-15 cells. Western blotting was used to assess the expression of RIG-I and MDA5 in control cells and PK-15 cells infected with PAstV-GX1 at an MOI of 0.01 for 24 h. The data are the means and standard deviations from three independent experiments. *, *P* < 0.05; **, *P* < 0.01.

The pattern recognition receptors (PRRs) RIG-I and MDA5 are usually activated by specific pathogen-associated molecular patterns (PAMPs). Enteroviruses, including poliovirus (PV), coxsackievirus B3 (CVB3), and enterovirus 71 (EV-D71), are single-stranded RNA (ssRNA) viruses that are positively sensed in the cytoplasm by MDA5 and RIG-I (38). As mentioned above, since the mRNAs of both RIG-I and MDA5 were activated after PAstV1 infection in PK-15 cells, we wanted to further determine whether RIG-I and MDA5 protein expression was also activated. As shown in Fig. 4, the protein expression levels of RIG-I and MDA5 were significantly increased compared to those of the control (*P* < 0.01), suggesting that PAstV1 induced the production of IFN-β by both RIG-I and MDA5 signaling pathways.

### Knockdown of RIG-I and MDA5 attenuated the production of IFN-β induced by PAstV1 in PK-15 cells.

**FIG 5:**
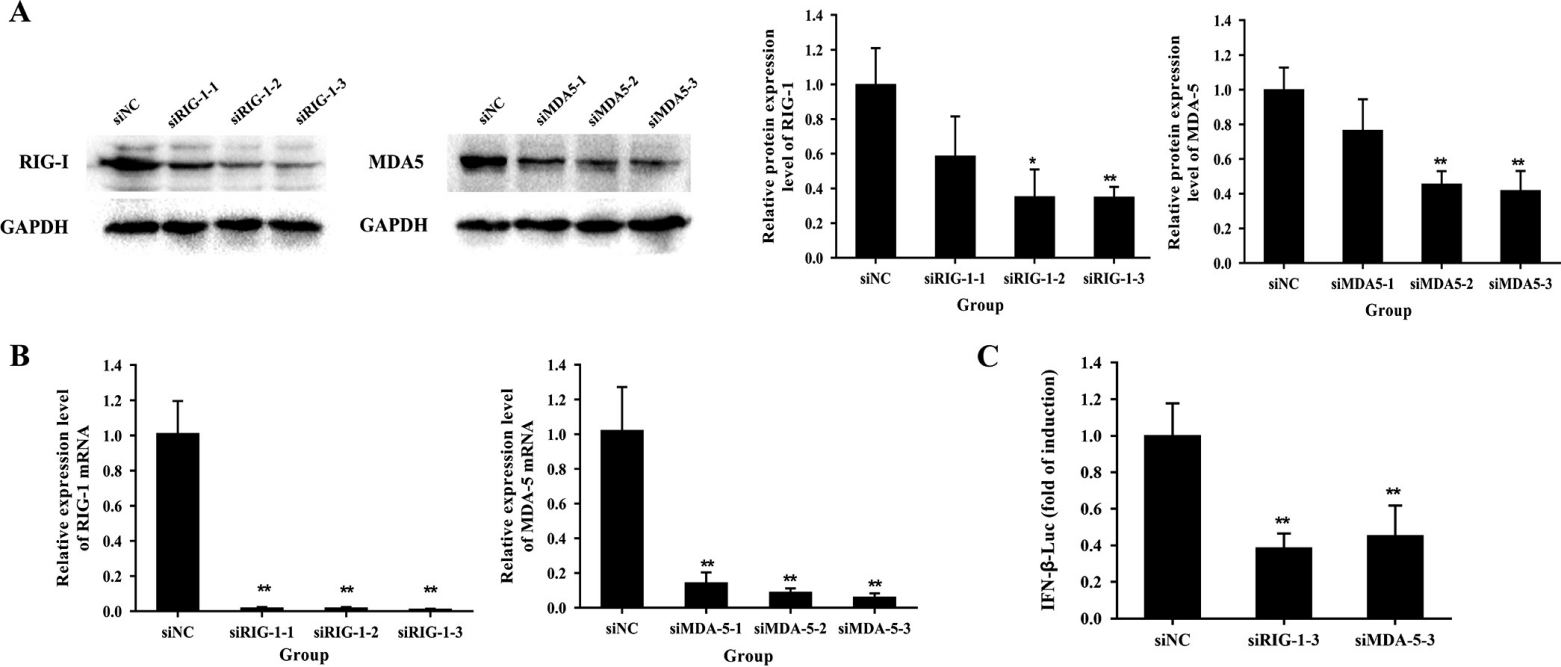
Changes in the activity of the IFN-β promoter in PK-15 cells infected with PAstV-GX1 after the knockdown of RIG-I and MDA5. (A and B) Western blotting (A) and quantitative RT-PCR (B) were used to measure the protein and mRNA expression levels of RIG-I or MDA5 in PAstV-GX1-infected PK-15 cells after transfection with siRIG-I-1, siRIG-I-2, and siRIG-I-3 or siMDA5-1, siMDA5-2, and siMDA5-3, respectively. (C) The activity of the IFN-β promoter in PAstV-GX1-infected PK-15 cells after transfection with siRIG-I-3 or siMDA5-3 was measured by using a luciferase assay. The data are the means and standard deviations from three independent experiments. *, *P* < 0.05; **, *P* < 0.01.

To further confirm the role of RIG-I and MDA5 in PAstV1-induced IFN-β production, three small interfering RNAs (siRNAs) for these molecules (siRIG-I and siMDA5) were designed to knock down their expression. As shown in Fig. 5A and B, siRIG-I-3 and siMDA5-3 significantly downregulated the expression of RIG-I and MDA5 at both the protein and mRNA levels compared to siRIG-I-1 and siMDA5-1, respectively (*P* < 0.01). Therefore, siRIG-I-3 and siMDA5-3 were selected for the subsequent experiments. In order to test the effects of RIG-I and MDA5 knockdown on interferon production, PK-15 cells were transfected with either siRIG-I-3 or siMDA5-3 for 6 h and subsequently infected with PAstV1 for 24 h. The levels of IFN-β promoter activity were then measured using the IFN-β promoter luciferase reporter gene assay. The results showed that the knockdown of RIG-I and MDA5 significantly reduced the activity of the IFN-β promoter compared to the control (*P* < 0.05) (Fig. 5C). These findings suggested that PAstV1 could induce the production of IFN-β via the RIG-I- and MDA5-mediated signaling pathways.

### Knockdown of RIG-I and MDA5 increased PAstV1 replication in PK-15 cells.

**FIG 6:**
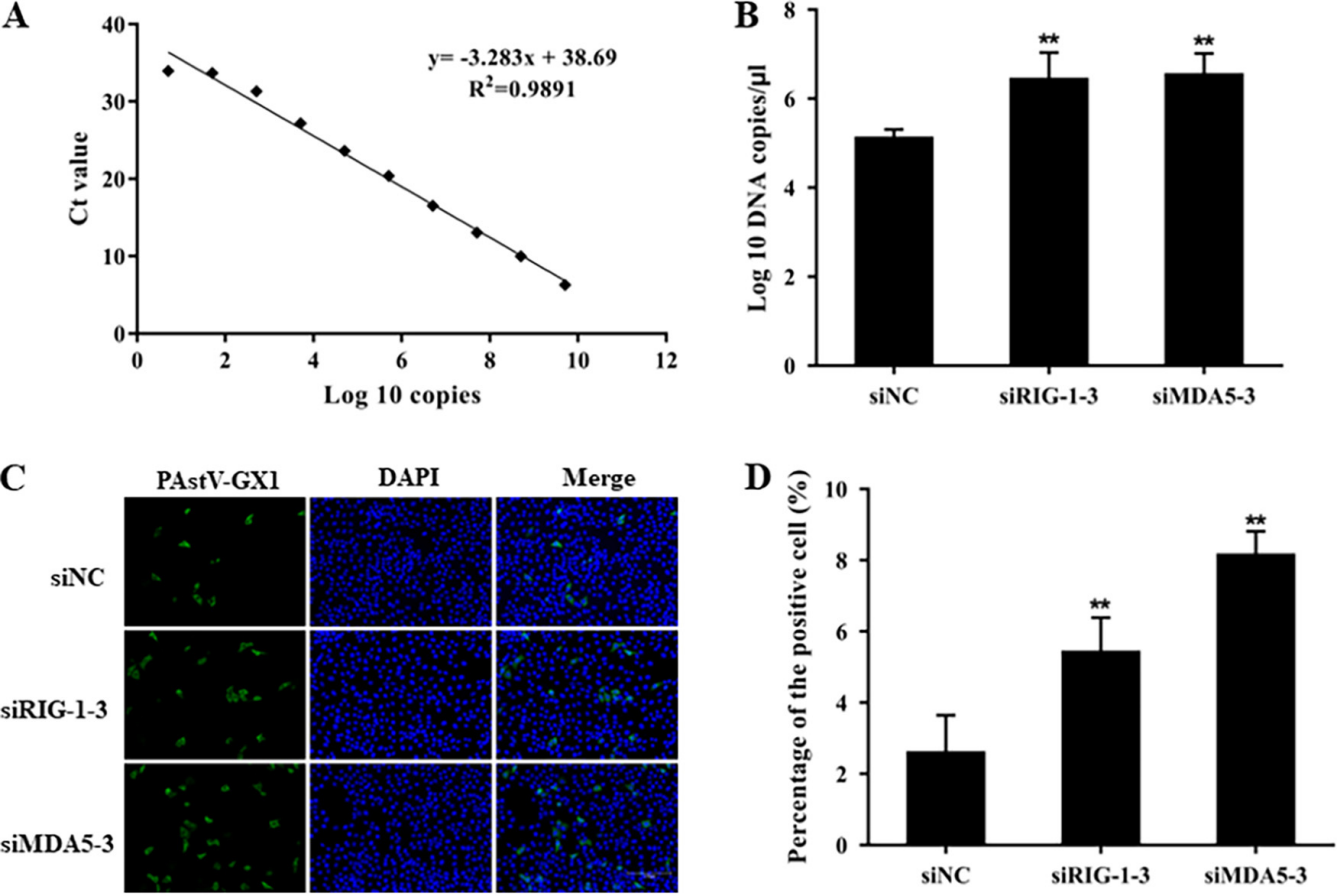
Changes in PAstV replication and viral loads in PK-15 cells after the knockdown of RIG-I and MDA5. (A) A 10-fold serial dilution of a known concentration of the PAstV1 plasmid was measured by quantitative RT-PCR, and a standard curve and a regression equation were established. The *x* axis shows the PAstV1 plasmid copy number as a log_10_ value, and the *y* axis indicates the corresponding cycle threshold (*C_T_*) value. (B) The viral loads in PAstV-GX1-infected PK-15 cells were measured by qPCR after transfection with the siRIG-I-3 or siMDA5-3 for 24 h. (C) PAstV-GX1 was stained with an anti-PAstV1 antibody (green), and the nuclei were stained with DAPI (blue). (D) Stained cells were observed under a fluorescence microscope, and 3 fields were randomly chosen to count the percentage of PAstV1-positive cells. The data are the means and standard deviations from three independent experiments. *, *P* < 0.05; **, *P* < 0.01.

Previous studies have shown that the addition of exogenous IFN-β to HAstV1-infected cells reduced viral replication (39). To determine whether endogenous IFN-β could also influence PAstV1 replication, the PAstV1 load and infection rate were measured in PK-15 cells by qPCR and an indirect immunofluorescence assay (IFA), respectively. The PAstV1 load was significantly higher in the siRIG-I-3 (*P* = 0.001) and siMDA5-3 (*P* = 0.024) treatment groups than in the control group (Fig. 6A and B). The IFA results also showed that the genome numbers and percentages of PAstV1-infected cells were significantly higher in the siRIG-I-3- and siMDA5-3-treated groups than in the control group (Fig. 6C). The percentages of PAstV1-positive cells in the negative-control and siRIG-I-3- and siMDA5-3-treated groups were 2.6% ± 1.3%, 5.4% ± 1.1%, and 8.2% ± 0.6%, respectively (Fig. 6D). These results demonstrated that IFN-β produced by PK-15 cells during PAstV1 infection inhibited PAstV1 replication.

## DISCUSSION

The innate immune response consists mainly of the production of IFNs and proinflammatory cytokines and chemokines, and it is an essential strategy for the prevention of host viral infection. Host cells perform this task via pattern recognition receptors (PRRs). In this study, we have examined the type I IFN response in PAstV1-infected PK-15 cells. Our findings demonstrate that PAstV1 infection induces a type I IFN response (Fig. 1A, B, and E) and that ISG15 and ISG56 mRNAs are detectable 12 h after infection with this virus (Fig. 3C and D). The increase in ISG mRNAs indicated that IFN-β was released into the extracellular matrix from PAstV1-infected cells. In previous work by Guix et al. using HAstV4, it was shown that IFN-β induction occurred late in infection, and this was found to be independent of replication (28). Here, we found that the levels of IFN-β were very low before 12 h after PAstV1 infection (Fig. 1A, B, and E), while the PAstV1 titers and the levels of viral gRNA were increased at 12 hpi (Fig. 1C and D). This indicated that IFN production occurred at the middle stages of the viral replication cycle and cytosolic RNA-sensing PRRs are initiating the pathway. Taken together, these results suggest that PK-15 cells can recognize invasion by PAstV1 and are able to induce innate immune responses.

Transcription factors involved in interferon production, including IRF3 and NF-κB, are activated when the virus is initially detected by the host cell. These activated transcription factors subsequently translocate to the nuclei and interact with the IFN promoter sequence, resulting in the upregulation of the IFN gene. In this study, after infection with PAstV-GX1, the addition of the IRF3 inhibitor resulted in a decrease in the mRNA expression level of IFN-β, while the mRNA levels of PAstV-GX1 were significantly increased (Fig. 2D and E). This finding was consistent with those of a previous study whereby cells were treated with 5 μM BX795, which resulted in a significant 2-fold increase in the total HAstV RNA produced as well as a significant increase in the amount of infectious progeny released into the supernatants (28). Moreover, Tam et al. found only low levels of NF-κB activation upon infection with HAstV compared to infections with adenovirus and human papillomavirus virus-like particles. In contrast, the IRF3 transcription pathways showed robust secretion of IFN-β (40). Combined with the above-described results, this suggests that NF-κB may mainly regulate the production of other cytokines, while the IRF3 transcription pathways are involved in the activation of IFN-β in host cells.

The viral recognition mediator leading to IFN production consists of a group of receptors located on the cytoplasmic and endosomal surfaces. These receptors include RIG-I, MDA5, and Toll-like receptors (TLRs). RIG-I is a cytoplasmic helicase that recognizes double-stranded RNA (dsRNA), and it is activated by retinoic acid, IFNs, as well as viral infections (41). MDA5 is a cytoplasmic virus sensor that recognizes ssRNA, and it relays signals that lead to IRF3 activation and, ultimately, IFN-β production (42). However, there are no studies regarding the recognition of IFN-producing PRRs by PAstV. In the present study, we confirmed that RIG-I and MDA5 were activated and that they contributed to the production of IFN-β in PAstV1-infected PK-15 cells. RIG-I and MDA5 mRNA and protein expression levels were upregulated at 12 and 24 hpi (Fig. 3A and B and Fig. 4), respectively, when the IFN-β expression level was high. IFN-β appears to be activated via RIG-I and MDA5 signaling. In this study, the siRNAs that were used to knock down RIG-I and MDA5 reduced the PAstV1-induced expression of RIG-I, MDA5, and IFN-β (Fig. 5). This finding suggests that RIG-I and MDA5 are essential for identifying the IFN produced, and there may be other signaling pathways involved in the production of IFN-β caused by PAstV1 infection. This will be further explored in future studies.

Many viruses have evolved mechanisms to evade the host’s innate immune response so that they gain a replication advantage in host cells. Several studies concluded that HAstVs may evade the immune system by preventing complement activation (40, 43–45). The complement system can eliminate pathogens, regulate the inflammatory response, and help shape the adaptive immune response. In addition, IFNs are the frontline defenders against viral infection, and their primary function is to locally restrict viral propagation (46). Two studies have explored the type I IFN response during HAstV infection (28, 39). These studies demonstrated that the type I IFN system can limit human and mouse AstV replication *in vitro* and *in vivo* and protects against AstV-induced barrier permeability (28, 39). The present study showed for the first time that the endogenous IFN-β induced in PAstV1-infected PK-15 cells may also limit the replication of this virus (Fig. 6). Furthermore, a recent study reported the rare isolation of a PAstV5 strain from tissue samples infected with clinical classical swine fever virus (CSFV) (47). CSFV coinfection was likely an important factor in the successful isolation of this strain by significantly enhancing its replication in PK-15 cells by suppressing the type I IFN response (47). Combined with the above-described results, we can infer that the type I IFN system can limit AstV replication *in vivo*.

Innate responses have been shown to play a role in controlling AstV replication in several animal models. In turkeys, AstV replication was shown to induce nitric oxide synthase in intestinal epithelial cells, suggesting that these cells are capable of enhancing their own defenses against viral infection (48). In mice, the levels of AstV replication in the intestine and viral shedding are significantly higher in Stat1^−/−^ animals than in wild-type mice (29). In piglets, PAstV infection upregulated the expression of IFN-β and ISG54 (19). Although we still do not know the molecular mechanisms by which PAstV evades innate immunity, we have confirmed that RLR transcription pathways are activated by PAstV1 infection. Future experiments in our laboratory will aim to explore the molecular mechanisms by which PAstV induces IFN at the cellular level.

In conclusion, PAstV was found to induce IFN-β production via the activation of RIG-I and MDA5 signaling pathways in PK-15 cells. Furthermore, the knockdown of RIG-I and MDA5 increased the production of IFN-β and enhanced the replication of PAstV. Our data will provide a useful reference for understanding the pathogenesis of AstVs. In addition, this study will help in the future development of effective anti-AstV drugs.

## MATERIALS AND METHODS

### Cells and viruses.

PK-15 cells were maintained in Dulbecco’s modified Eagle’s medium (DMEM; Gibco, USA) containing 10% heat-inactivated fetal bovine serum (FBS; Gibco, USA) at 37°C in a 5% CO_2_ incubator.

The PAstV type 1 strain, named PAstV-GX1 (GenBank accession no. KF787112), was isolated from a PAstV-positive fecal sample from a diarrheal pig in a farm in Nanning, Guangxi Province, China, in 2013 (19). The PAstV-GX1 strain was stably passaged in PK-15 cells and cultured in DMEM containing 0.5 μg/mL tosyl-l-phenylalanine chloromethyl ketone (TPCK)-treated trypsin (Sigma-Aldrich, Germany). The PAstV1 stock titers were 1 × 10^7.3^ 50% tissue culture infective doses (TCID_50_)/mL.

### Real-time quantitative PCR.

The total RNA was extracted from the tissue and fecal samples by using the EZNA HP total RNA kit (Omega Biotech, Doraville, GA, USA). Next, 300 ng of RNA extraction products from each sample was reverse transcribed using a reverse transcription (RT) kit (Vazyme, Inc., Nanjing, China) according to the manufacturer’s instructions. A thermocycler (LightCycler 96; Roche) was used for quantitative PCR. The sequences were obtained from the National Center for Biotechnology Information (NCBI), and Primer 5.0 software was used for designing the related primers (Table 1).

**TABLE 1:** Primer sequences used for real-time quantitative PCR

Primer	Sequence	
	Forward (5′–3′)	Reverse (5′–3′)
po-IFN-β	AGTGCATCCTCCAAATCGCT	GCTCATGGAAAGAGCTGTGGT
po-RIG-I	AGAGCAGCGGCGGAATC	GGCCATGTAGCTCAGGATGAA
po-MDA-5	CAGTGTGCTAGCCTGCTCTG	GCAGTGCCTTGTTTCCTCTC
po-ISG-15	CGCAGCAGCCCCTATGAG	GACAGCCAGAACTGGTCTGCTT
po-ISG-56	AAATGAATGAAGCCCTGGAGTATT	AGGGATCAAGTCCCACAGATTTT
po-β-actin	GTGATCTCCTTCTGCATCCTGTC	GCAAGAACTCACAGGACAGGAA
PAstV-1	ATCAACTCTAAACCAGGAGCGAACG	TTGGACCTGTGACACCTGATTTG

The full-length cDNA of PAstV--GX1 was cloned into T-Vector, and named PAstV1. (13). Three biological replicates and technical replicates were performed in each experiment.

### Transfection and luciferase reporter assays.

PK-15 cells plated into 12-well plates were transfected with reporter plasmid pIFN-β-luc (350 ng) and renilla luciferase plasmid pRL-TK (50 ng). The transfections were performed by using Lipo8000 transfection reagent (Beyotime, Inc., Shanghai, China) according to the manufacturer’s instructions. At 6 h posttransfection, the cells were infected with PAstV1 at a multiplicity of infection (MOI) of 0.1 for 1 h. After 1 h of incubation, the cells were washed with phosphate-buffered saline (PBS) and incubated in DMEM containing 0.5 μg/mL TPCK. Cell lysates were collected 4, 8, 12, 24, and 48 h after infection, and the activity of the IFN-β promoter was measured using a dual-luciferase reporter gene assay kit (Bioscience, Inc., Shanghai, China). The relative firefly luciferase activity was normalized to the renilla luciferase activity. A microplate reader (Spark 10M; Tecan, Switzerland) was used to measure the luminescence signals obtained.

### IFN bioassay.

To measure the effect of PAstV1 on the amount of IFN produced by PK-15 cells, conditioned media (500 μL) from PAstV-infected PK-15 cells were UV treated to inactivate infectious PAstV and overlaid onto PK-15 cells seeded in a 24-well plate. After 24 h of treatment, the PK-15 cells were infected with IFN-sensitive VSV-GFP at an MOI of 0.1. Twenty-four hours after infection, viral replication was assessed by fluorescence microscopy at excitation and emission wavelengths of 488 and 507 nm, respectively.

### Pharmacological inhibition assay.

To examine the levels of toxicity of the NF-κB inhibitor BAY11-7082 (Beyotime, Inc., Shanghai, China) and the IRF3 inhibitor BX795 (MedChemExpress, USA) on PK-15 cells, we performed an MTT-based cytotoxicity assay. Briefly, PK-15 cells were seeded into 96-well plates at 1 × 10^4^ cells/well. When the cells were 70% confluent, they were incubated with different concentrations of either BAY11-7082 or BX795 for 72 h. The medium was removed, and the cells were washed twice with PBS. Next, 10 μL of an MTT solution was added to each well, and the cells were incubated at 37°C for a further 4 h. The medium was removed, 150 μL of dimethyl sulfoxide (DMSO) was added to each well, and the cells were incubated in the dark for 10 min. Cell viability was measured spectrophotometrically at 570 nm using a microplate reader (Spark 10M; Tecan, Switzerland). The optical density (OD) values were normalized to those of the control group.

To examine the levels of the inhibitory effects of the NF-κB inhibitor BAY11-7082 and the IRF3 inhibitor BX795 on PK-15 cells, we performed a Western blot assay. Briefly, PK-15 cells were seeded into 12-well plates at 1 × 10^5^ cells/well. When the cells were 70% confluent, they were incubated with either 5 μM BAY11-7082 or 0.5 μM BX795 and infected with PAstV-GX1 at an MOI of 0.01 for 24 h. Subsequently, nuclear proteins or cytosolic proteins were isolated separately using a nuclear protein extraction kit (Solarbio, Inc., Beijing, China).

The PK-15 cells were divided into 4 groups: (i) the negative-control group, (ii) the PAstV1 group, (iii) the BAY11-7082 treatment group, and (iv) the BX795 treatment group. At 70% cell confluence, the BAY11-7082 and BX795 treatment groups were pretreated with 5 μM BAY11-7082 and 0.5 μM BX795 for 1 h, respectively. Subsequently, the cells were washed with PBS and incubated with PAstV1 at an MOI of 0.01 (excluding the negative-control group). After 1 h of incubation, the cells were washed with PBS, and the BAY11-7082- and BX795-treated groups were incubated in DMEM containing 0.5 μg/mL TPCK and 5 μM BAY11-7082 or 0.5 μM BX795, respectively. The negative-control and PAstV1 groups were incubated in DMEM containing 0.5 μg/mL TPCK only. The cells were collected at 24 h for the detection of IFN-β and PAstV-GX1 mRNAs.

### Western blotting.

The total protein was extracted from PK-15 cells using radioimmunoprecipitation assay (RIPA) lysis buffer with 1% phenylmethanesulfonyl fluoride and a phosphatase inhibitor (Cwbio, Inc., Jiangsu, China). Following centrifugation at 12,000 × *g* for 15 min, the concentration of the total protein was quantified using a bicinchoninic acid assay kit (Beyotime, Inc., Shanghai, China), and loading buffer was added to the protein samples for denaturation at 98°C for 15 min. Each lane was loaded with the same amount of protein (30 μg) for sodium dodecyl sulfate-polyacrylamide gel electrophoresis, and the proteins were then transferred onto a polyvinylidene difluoride (PVDF) membrane at 250 V for 30 min. After the transfer, 5% bovine serum albumin (BSA) dissolved in PBS-Tween (PBST) was used for blocking at 25°C for 1 h. The membranes were then incubated with antibodies to p-IRF3 (Cell Signaling Technology, USA), RIG-I (Abmart, Inc., Shanghai, China), MDA5 (Abmart, Inc., Shanghai, China), p65 (Abmart, Inc., Shanghai, China), β-actin (Beyotime, Inc., Shanghai, China), glyceraldehyde-3-phosphate dehydrogenase (GAPDH) (Beyotime, Inc., Shanghai, China), and histone 3 (Beyotime, Inc., Shanghai, China) diluted 1:1,000 in PBS for 16 h at 4°C. The membranes were then washed three times with Tris-HCl-buffered saline with Tween 20 (TBST). The corresponding peroxidase-conjugated secondary antibodies were then added, and the membranes were incubated at 37°C for 1 h. Luminous fluid (Cowin Biotech Co., Ltd., Jiangsu, China) was used to detect the proteins on the membrane, and Image Lab software (Bio-Rad, Inc., USA) was used for gray analysis of bands on the membrane.

### RNA interference.

Gene sequences of RIG-I and MDA5 were obtained from the NCBI GenBank database. Small interfering RNA (siRNA) sequences targeting these genes were designed using the BLOCK-iT RNAi (RNA interference) designer. A negative-control siRNA (siNC) and three target sequences for RIG-I or MDA5 were selected to construct the siRNAs, and these were designated siRIG-I-1, siRIG-I-2, and siRIG-I-3 or siMDA5-1, siMDA5-2, and siMDA5-3, respectively (Table 2). The sense and antisense siRNAs were synthesized by Genepharma (Suzhou, China). To determine the transfection efficiency, PK-15 cells were seeded at 1 × 10^5^ cells/well in a 12-well plate. Upon reaching 50% cell confluence, the cells were transfected with siRNA at 40 pmol/well using Lipo8000 transfection reagent (Beyotime, Inc., Shanghai, China) according to the manufacturer’s instructions.

**TABLE 2:** Small interfering RNA sequences used for RIG-I and MDA5 knockdown experiments

siRNA	Sense sequence (5′–3′)	Antisense sequence (5′–3′)
siNC	UUCUCCGAACGUGUCACGUTT	ACGUGACACGUUCGGAGAATT
siRIG-I-1	GCCCUUAACCAAGCAGGUUTT	AACCUGCUUGGUUAAGGGCTT
siRIG-I-2	GCAAACAGCAUCCUUAUAATT	UUAUAAGGAUGCUGUUUGCTT
siRIG-I-3	CCAUAACUCUUGGAGGCUUTT	AAGCCUCCAAGAGUUAUGGTT
siMDA5-1	GCUAUCUCAUCUCGUGUUUTT	AAACACGAGAUGAGAUAGCTT
siMDA5-2	GCACUUGCCCGCGAAUUAATT	UUAAUUCGCGGGCAAGUGCTT
siMDA5-3	GCAGAUUCUUCUGUAGUUUTT	AAACUACAGAAGAAUCUGCTT

The PK-15 cells were divided into the following three groups: (i) the siNC group, (ii) the siRIG-I group, and (iii) the siMDA5 group. Upon reaching 70% confluence, the PK-15 cells were transfected with siNC, siRIG-I-3, and siMDA5-3 for 6 h. Subsequently, the cells were incubated with PAstV1 at an MOI of 0.01 for 1 h. Following infection, the cells were washed with PBS and incubated in DMEM containing 0.5 μg/mL TPCK. The replication of PAstV1 and the expression levels of IFN-β were measured 24 h after infection.

### Immunofluorescence analysis.

To fix the PK-15 cells, cold acetone was applied for 30 min at −20°C, followed by five washes with PBS. PBS containing 0.5% Triton X-100 was used to permeabilize the cells for 20 min, and 5% BSA was then used to block the cells for 1 h. PAstV1 Cap-specific antibodies (49) were then added, and the cells were transferred to 4°C for 16 h. After five more washes with PBS, anti-mouse IgG(H+L)–Alexa Fluor 488 (Abmart, Inc., Shanghai, China) was added, and the cells were incubated for 1 h at 25°C. 4′,6-Diamidino-2-phenylindole (DAPI; Servicebio, China) was used for nuclear staining. The localization of PAstV1 in PK-15 cells was observed and imaged using a fluorescence microscope (Thermo, USA).

### Statistical analysis.

Differences between the experimental and control groups were analyzed using GraphPad Prism software by one-way analysis of variance followed by a least-square-difference multiple-comparison test. Data are expressed as the means ± standard deviations (SDs). Statistical significance is indicated with asterisks in the figures (*, *P* < 0.05; **, *P* < 0.01; ***, *P* < 0.001). Unless indicated otherwise, the experiments were performed in triplicate (*n* = 3).
